# The immune checkpoint regulator PD-L1 expression are associated with clinical progression in prostate cancer

**DOI:** 10.1186/s12957-021-02325-z

**Published:** 2021-07-16

**Authors:** Juan He, Min Yi, Lingfeng Tan, Jianghua Huang, Lin Huang

**Affiliations:** 1 Department of Pathology, The First Affiliated Hospital of Guangxi Medical University, Nanning, China; 2 Department of Pathology, The Four Affiliated Hospital of Guangxi Medical University, Liuzhou, China; 3 Department of Urology, The First Affiliated Hospital of Guangxi Medical University, No. 6 Shuangyong Road, Guangxi Zhuang Autonomous Region, Nanning, 530021 China

**Keywords:** PD-L1, PD-1, Prostate cancer, Relationship

## Abstract

**Background:**

The programmed death 1 (PD-1)/programmed death-ligand 1 (PD-L1) have shown positive efficacy in several solid cancers due to their targeted antitumour effects. However, the frequency and clinical implication value in prostate cancer still remain unclear.

**Methods:**

The PD-1/PD-L1 expression was detected by immunohistochemical (IHC) analysis in 96 retrospectively collected cases of prostatic cancer and 44 controls of benign prostatic hyperplasia (BPH). Its correlation with clinicopathological features including age, PSA level, Gleason score, lymph node metastasis, clinical T stage and risk factor grade in prostate cancer was also assessed.

**Results:**

The PD-L1-positive expression was significantly higher in cancer cases compared with benign tissues, whereas no difference in PD-1 positive expression was found. Moreover, the PD-L1 expression in tumour cells or lymphocytes was associated with Gleason score, but not related to age, preoperative PSA level, clinical T-stage, lymph node metastasis and grade of risk factors. In addition, no association between the positive expression of PD-1 and PD-L1 in tumour cells and lymphocytes was found.

**Conclusions:**

The expression of PD-L1 not PD-1 is highly prevalent in prostate cancer. PD-L1 is closely related to Gleason score and may be a co-factor associated with the progression of prostate cancer.

## Introduction

Prostate cancer has become the second most common malignant cancer among men, with approximately 174,650 new cases occurred in America [1]. Nowadays, besides surgery, radiotherapy and chemotherapy, immunotherapy involving PD-1/PD-L1 inhibitors has become a new promising treatment in the field of cancer therapy. PD-1/PD-L1 inhibitors can suppress the adaptive immune system and reverse the mechanism of tumour immune escape by blocking the PD-1/PD-L1 signal pathway; therefore, the immune system can recover and kill tumour cells directly [2–5]. Sfanos et al. [6] found that the overexpression of PD-1 on CD8+ T cells in the prostate cancer microenvironment induced the failure of these CD8+ T cells to produce the corresponding anticancer response. Meanwhile, the results of animal experiments also showed that the efficacy and prognosis of immunotherapy had a correlation with the expression of PD-1/PD-L1 on relevant CD8+ T cells in prostate cancer. The combination of PD-1/PD-L1 inhibitors could significantly prolong the disease-free progression survival period of animals in the experimental group.

Remarkably, with the development of tumour immunotherapy, the role of PD-1/PD-L1 inhibitors has attracted more attention. In clinical treatment, PD-1/PD-L1 inhibitors combined with androgen receptor antagonists can improve the effect and prognosis of tumour treatment [7–9]. Graff et al. [8] used PD-1 inhibitors to carry out a phase II clinical trial treatment of castrated resistant prostate cancer (mCRPC). The results showed that 3 of the 10 patients included in the study showed obvious anti-tumour reactions and no immune-related adverse reactions. Similarly, Bishop et al. [9] had shown ENZ resistance CRPC is associated with a high frequency of PD-1/L1 therapy targets, not only in the mouse models, but in patients.

Previous studies detecting the positivity of PD-L1 expression (and/or PD-1 in a few reports) in prostate cancer specimens had yielded variable results. Additionally, further studies are needed, as few data in BPH and limited studies assessing the clinicopathological significance were associated with the expression of PD-1/PD-L1 in prostate cancer. Herein, our study aimed to retrospectively assess the PD-1/PD-L1 expression status in prostate cancer and BPH tissue by immunohistochemistry, as well as the association between PD-1/PD-L1 and related clinicopathological parameters including age, PSA, Gleason score, lymph node metastasis, clinical T stage and risk factor grade.

## Materials and methods

### Patient characteristics and clinicopathological data

Ninety-six prostate cancer tissue specimens were obtained retrospectively from patients performed with RP or transrectal ultrasound-guided prostatic biopsy in the Urology Department of The Fourth Affiliated Hospital of Guangxi Medical University between 2012 and 2015. These patients were diagnosed by two senior pathologists. The mean age of prostate cancer patients was 70 years (72.1 ± 6.4) (range 42–78 years). None of them received surgical castration, drug castration, radiotherapy or chemotherapy before the operation. According to the EAU urological disease diagnosis guidelines, patients were divided into the following groups: (1) age < 60, 60–69, 70–79 and > 80 (years); (2) the serum TPSA value: < 4, 4–10, 10–20 and > 20 (ng/ml); (3) Gleason score < 7, 7 and > 7; (4) clinical stage T1 + T2 and T3 + T4 groups; and (5) grade of risk factors: low-risk group (PSA < 10 ng/ml, Gleason score < 7, T ≤ T2a), medium risk group (PSA, 10–20 ng/ml, Gleason score = 7, T = T2b) and high-risk group (PSA > 20 ng/ml, Gleason score ≥ 8, T ≥ T2c). In the control group, 44 BPH patients were collected from the same period. The mean age was 70.6 ± 6.9 years. There was no significant difference in age between the two groups. Our study was approved by the Institutional Ethics Review Board of The Fourth Affiliated Hospital of Guangxi Medical University. All participants signed an informed consent for the use of the samples during hospitalisation. The clinical data of all these participants were retrospectively obtained from the hospital electronic patient record system.

### Immunohistochemistry

Four-micrometre paraffin-embedded prostate tissue sections were dewaxed and then washed with PBS. We repaired on the slide used antigen repair buffer EDTA (MVS-0098, Fuzhou Maixin Biotechnology Development Co., Ltd.). Blocking was performed by hydrogen peroxide (H44024859, Guangdong Nanguo Pharmaceutical Co., Ltd.) for 10 min at room temperature. The sections were then incubated overnight with the anti-PD-1 antibody (mouse anti-human, #MAB-0654, Fuzhou Maixin Biotechnology Development Co., Ltd.) or the anti-PD-L1 antibody (mouse anti-human, #RMA-0732, Fuzhou Maixin Biotechnology Development Co., Ltd.), the anti-CD3 antibody (rabbit anti-human, #1912110543a, Fuzhou Maixin Biotechnology Development Co., Ltd.), the anti-CD4 antibody (mouse anti-human, #2005270620c, Fuzhou Maixin Biotechnology Development Co., Ltd.), the anti-CD8 antibody (rabbit anti-human, #2005130514b, Fuzhou Maixin Biotechnology Development Co., Ltd.), the anti-CD68 antibody (mouse anti-human, #2010280041e, Fuzhou Maixin Biotechnology Development Co., Ltd.) and the anti-CD163 antibody (mouse anti-human, #2102030206a, Fuzhou Maixin Biotechnology Development Co., Ltd.) at 4 °C and incubated with secondary antibody (MaxVision-HRP, Fuzhou Maixin Biotechnology Development Co., Ltd.) for 1 h at room temperature. Peroxidase activity was detected using the DAB reagent kit (× 20) (DAB-1031, Fuzhou Maixin Biotechnology Development Co., Ltd.). The nuclei were counterstained with haematoxylin (Lot: 180301, Shanghai Biological Technology Development Co., Ltd.).

### Evaluation of immunohistochemistry

All stains were analysed independently by two pathologists. Representative viable tissue sections were scored semi-quantitatively for staining status as follows: weak staining (light yellow), moderate staining (dark yellow) and strong staining (brown). The positive PD-1/PD-L1 expression was defined as when at least 1% of tumour cells/lymphocytes were seen with moderate to strong staining or at least 10% of tumour cells/lymphocytes were seen with weak staining [10]. As for lymphocytes/macrophages, representative viable tissue sections were scored semi-quantitatively for density as follows: (1) 1 (0–10% cells), (2) 2 (11–50% cells), (3) 3 (51–75% cells) or (4) 4 (75–100% cells) per 0.6-mm tissue core. Staining status was as follows: (1) 0 (not stained), (2) 1 (light yellow), (3) 2 (dark yellow) or (4) 3 (brown). If the score of staining status and proportion of positive cells is less than or equal to 4, it indicates negative expression. If the score is higher than 4, it indicates positive expression.

### Statistical analysis

Statistical analyses were performed using the statistical software IBM SPSS, version 17.0 (SPSS Inc. Chicago, IL). The differences between the case and control groups and the associations between the PD1/PDL1 expression and the clinicopathological parameters of prostate cancer patients were analysed by chi-square test or Fisher’s exact test. All statistical analyses were two-sided, and *P* < 0.05 was considered to be statistically significant level.

## Results

### Expression of PD-1 and PD-L1 in prostate tissue

**Fig. 1:**
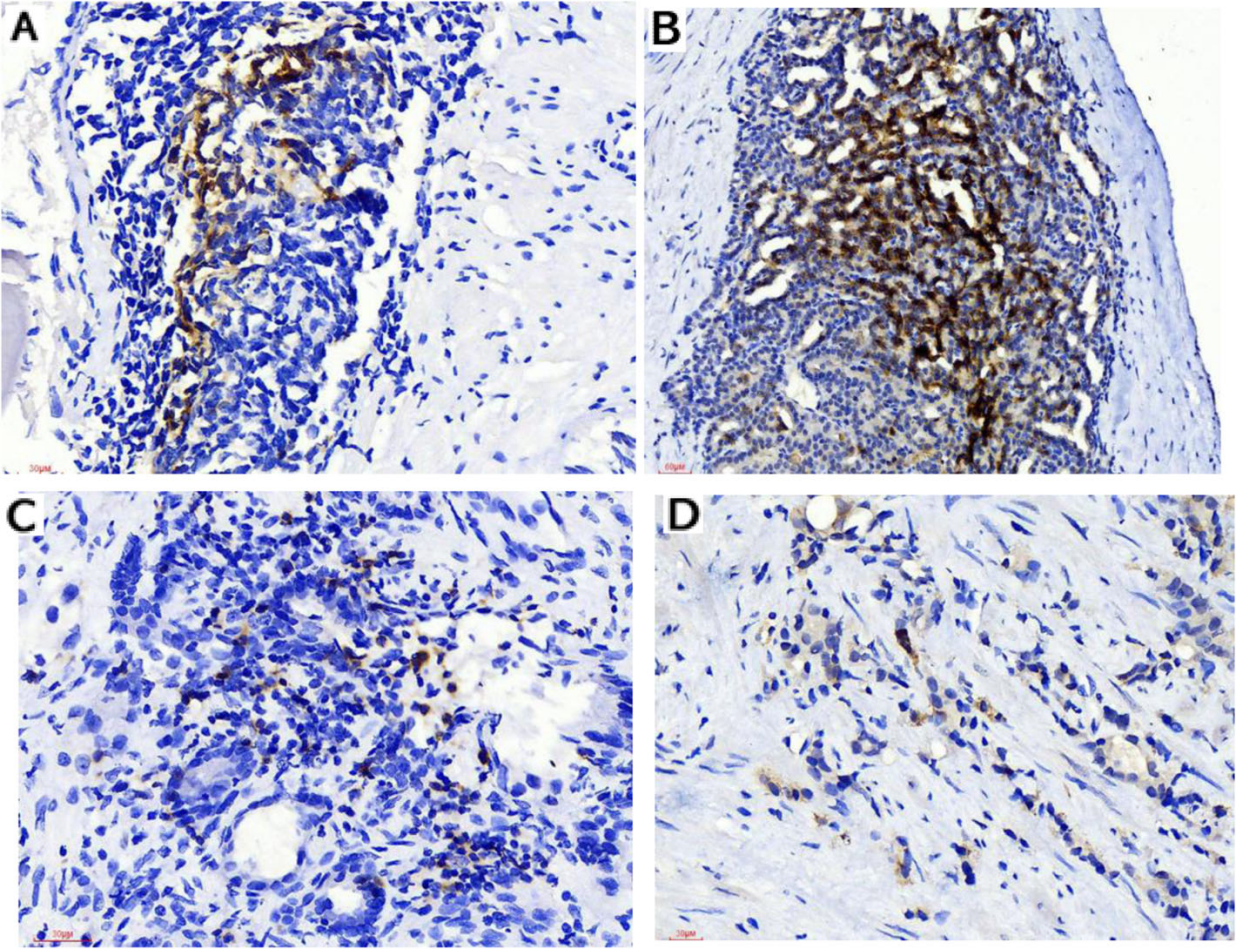
Representative immunohistochemical (IHC) staining of PD1 and PDL1 in prostate cancer tissues. **A**, **C** Representative images showing PD-1 expression in tumour epithelial cells and lymphocytes, respectively. **B**, **D** Representative images showing PD-L1 expression in tumour epithelial cells and lymphocytes, respectively. Original magnification, × 400. PD-1, programmed cell death protein 1; PD-L1, programmed death-ligand 1

**Fig. 2:**
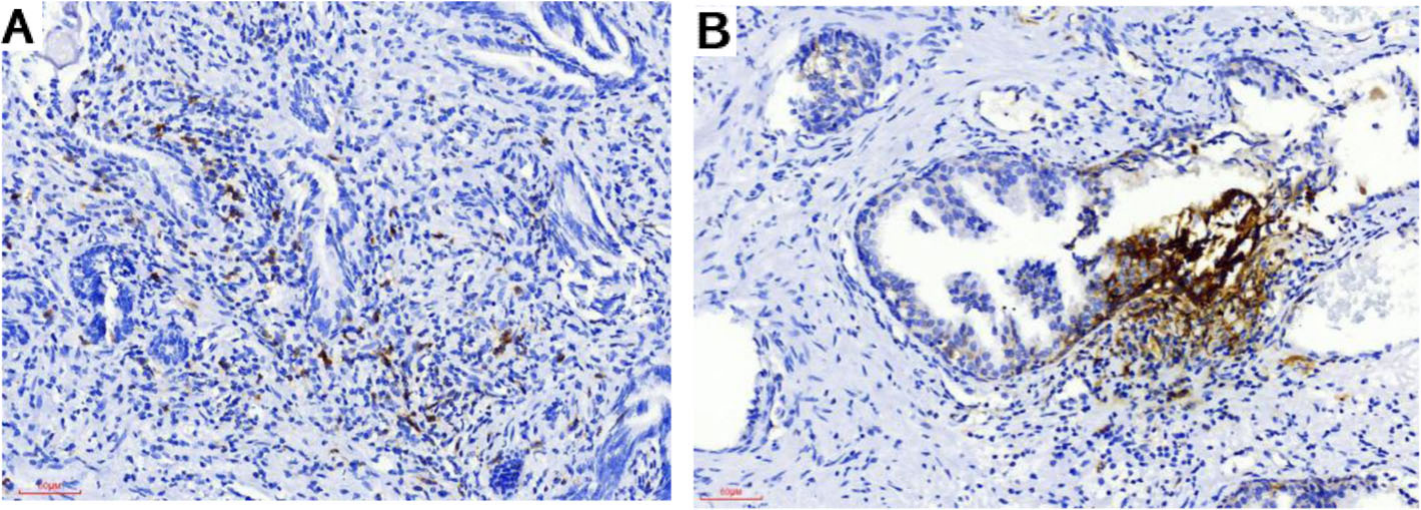
Representative immunohistochemical (IHC) staining of PD1 and PDL1 in BPH. **A** Representative image showing PD-1 expression in lymphocytes. **B** Representative image showing PD-L1 expression in epithelial cells and lymphocytes. Original magnification, × 200. PD-1, programmed cell death protein 1; PD-L1, programmed death-ligand 1; BPH, benign prostatic hyperplasia

In total, 96 cases of prostatic carcinoma and 44 controls of prostatic hyperplasia were immunohistochemically stained for PD-1 and PD-L1. Representative immunohistochemical staining is shown in Figs. 1 and 2. In prostatic carcinoma tissue, positive staining of PD1/PDL1 was seen in the cytoplasm of the epithelial cells and lymphocytes (Fig. 1). Similar to prostatic carcinoma tissue, positive staining for PD1/PDL1 was also detected in the cytoplasm of the epithelial cells and lymphocytes in benign tissues (Fig. 2).

### The positive expression rate of PD-1 and PD-L1 in prostate cancer compared with BPH

The expression of PD-1 and PD-L1 in epithelial cells was positive in 2 (2.1%) and 24 (25.0%) of the 96 cancer cases, and in 1 (2.2%) and 2 (4.5%) of the 44 benign tissues, respectively. Meanwhile, PD-1- and PD-L1-positive expression in lymphocytes cells were seen in 13 (13.5%) and 26 (27.1%) cases and also in 2 (4.5%) and 5 (11.4%) of benign tissues, respectively. Thus, the positive rate of PD-L1 expression was significantly higher in cancerous than in benign tissues, while no significant difference of PD-1-positive expression was found (Table 1). In addition, no association was found between PD-1-positive expression in tumour cells and lymphocytes (*P* = 0.128; Table 2) or with PD-L1 (*P* = 0.185; Table 3).

**Table 1:** PD-1 and PD-L1 status of prostate cancer cases and BPH controls (IMC)

	PCa, *n* = 96 (%)	BPH, *n* = 44 (%)	*X*^2^	*P*
PD-1 epithelial positive (%)	2 (2.1)	1 (2.2)	0.000	1.000
PD-L1 epithelial positive (%)	24 (25.0)	2 (4.5)	7.050	0.008
PD-1 lymphocytes positive (%)	13 (13.5)	2 (4.5)	1.899	0.192
PD-L1 lymphocytes positive (%)	26 (27.1)	5 (11.4)	4.325	0.038

*PD-1* programmed cell death protein 1, *PD-L1* programmed death-ligand 1

**Table 2:** PD-1 in tumour cells versus lymphocytes

	PD-1 in lymphocytes		
	Negative	Positive	*P*
PD-1 negative in tumour cells	82	12	0.128
PD-1 positive in tumour cells	1	1	

*PD-1* programmed cell death protein 1

**Table 3:** PD-L1 in tumour cells versus lymphocytes

	PD-L1 in lymphocytes		
	Negative	Positive	*P*
PD-L1 negative in tumour cells	55	17	0.185
PD-L1 positive in tumour cells	15	9	

*PD-L1* programmed death-ligand 1

### Lymphocyte infiltration and correlations

**Fig. 3:**
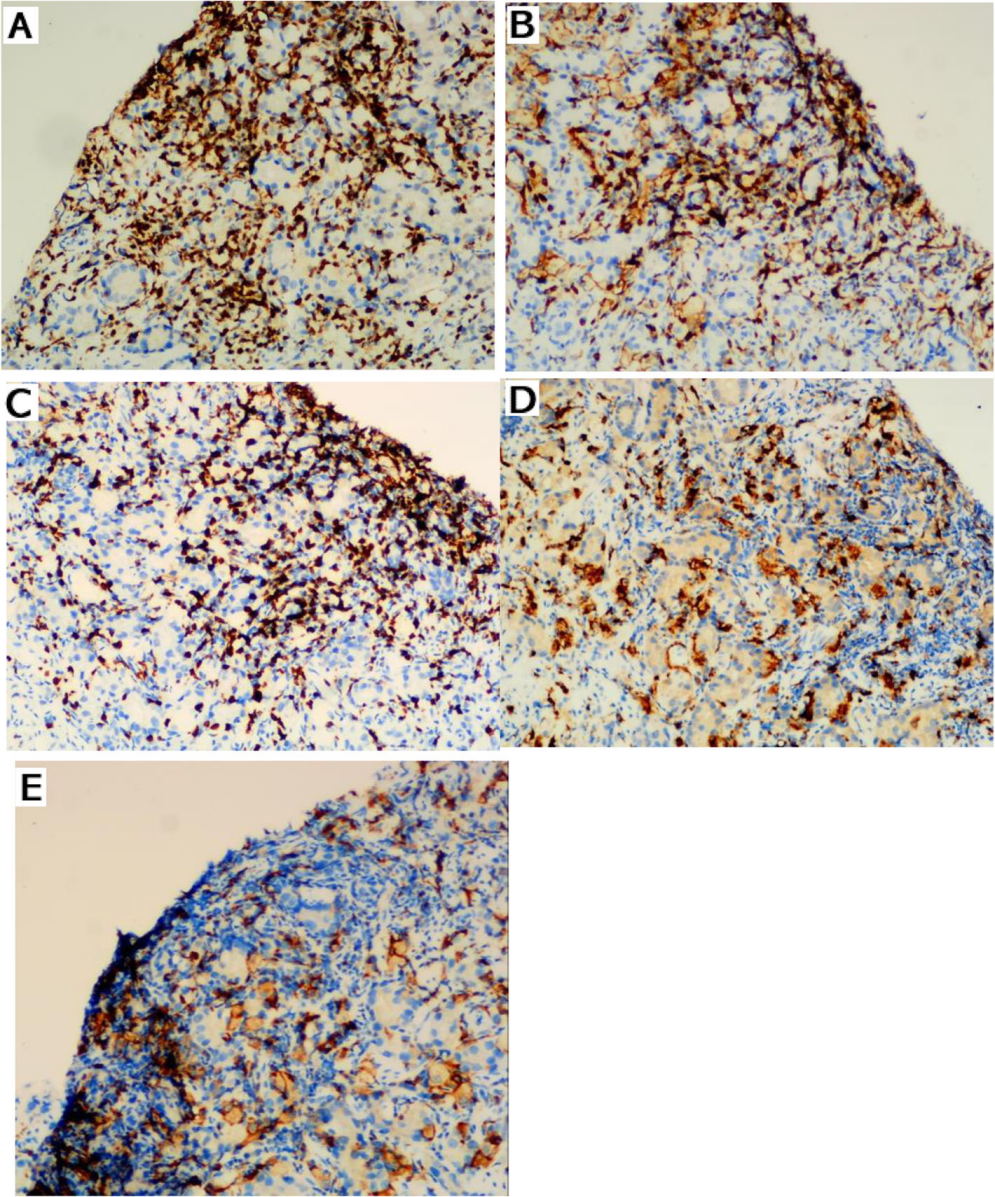
Representative immunohistochemical (IHC) staining of CD3, CD4, CD68 and CD163 in prostate cancer tissues. **A** Representative images showing CD3 expression. **B** Representative images showing CD4 expression. **C** Representative images showing CD8 expression. **D** Representative images showing CD68 expression. **E** Representative images showing CD163 expression. Original magnification, × 200

We performed CD3 marking in the ninety-six prostate cancer tissue specimens and relativized them by the expression of PD-L1. No association between the PD-L1-positive expression and CD3+ lymphocytes was shown (*P* = 0.607; Table 4). Subsequently, tumour infiltrating lymphocytes and macrophages were observed microscopically in 26 PD-L1-positive lymphocyte prostate cancer tissues. We identified the lymphocytes showing the expression of the markers CD4 and CD8 to differentiate the lymphocyte lineage. We also identified macrophages of the M1 and M2 lineages with markers of CD68 (M1) and CD163 (M2), but no statistically significant difference was observed between the expression of CD4+ T cells and CD8+ T cells, or for M1 and M2 macrophages (Table 5). Representative immunohistochemical staining is shown in Fig. 3. The positively stained cells were seen in the cytoplasm of the lymphocytes.

**Table 4:** PD-L1 in lymphocytes versus CD3+ cells

	PD-L1 in lymphocytes		
	Negative	Positive	*P*
CD3 negative	14	4	0.607
CD3 positive	56	22	

*PD-L1* programmed cell death protein L1

**Table 5:** The CD4, CD8, CD68 and CD163 expression in 26 PD-L1-positive lymphocytes cases

	Negative	Positive	*P*
CD4	4	22	0.308
CD8	7	19	
CD68	8	18	0.388
CD163	11	15

### Correlation between PD-1/PD-L1 expression and clinicopathological parameters of prostate cancer cases

In order to evaluate the correlation between PD-1/PD-L1 expression and clinicopathological parameters of prostate cancer cases, we compared the expression of PD-1/PD-L1 in each clinicopathological parameter group of prostate cancer cases. The PD-1/PD-L1 expression in tumour cells and lymphocytes and their correlations with clinicopathological characteristics in prostate cancer cases are summarised in Tables 6 and 7. The results showed that the PD-L1 expression in tumour cells or lymphocytes was associated with Gleason score, but it was not related to age, preoperative PSA level, clinical T-stage, lymph node metastasis and grade of risk factors, whereas no statistically significant associations were seen between PD-1 expression in tumour cells or tumour-infiltrating lymphocytes and age, PSA level, Gleason score, clinical T-stage, lymph node metastasis and grade of risk factors.

**Table 6:** PD-1/PD-L1 expression in tumour cells

Variable	Number	PD-1			PD-L1		
		Negative	Positive	*P*	Negative	Positive	*P*
**Age**				0.175			0.320
< 60	5	5	0		2	3	
60–69	25	25	0		19	6	
70–79	38	38	0		29	9	
> 80	28	26	2		22	6	
**PSA**				0.895			0.270
< 4.0	5	5	0		2	3	
4–10	3	3	0		2	1	
10–20	14	14	0		10	4	
> 20	74	72	2		58	16	
**Gleason score**				0.719			0.049
**< 7**	5	5	0		5	0	
7	25	24	1		23	2	
≥ 8	66	65	1		44	22	
**pT stage**				0.095			0.204
T1+T2	30	28	2		20	10	
T3+T4	66	66	0		52	14	
**pN stage**				0.180			0.404
pN0	41	39	2		29	12	
pN1	55	55	0		43	12	
**Grade of risk factors**				1.000			0.638
Moderate	6	6	0		4	2	
High	90	88	2		68	22

**Table 7:** PD-1/PD-L1 expression in tumour-associated lymphocytes

Variable	Number	PD-1			PD-L1		
		Negative	Positive	*P*	Negative	Positive	*P*
**Age**							
< 60	5	3	2	0.164	3	2	0.880
60–69	25	20	5		19	6	
70–79	38	35	3		27	11	
> 80	28	25	3		21	7	
**PSA**				0.215			0.376
< 4.0	5	3	2		3	2	
4–10	3	2	1		1	2	
10–20	14	12	2		11	3	
> 20	74	66	8		55	19	
**Gleason score**				0.613			0.034
**< 7**	5	4	1		5	0	
7	25	23	2		22	3	
≥ 8	66	56	10		43	23	
**pT stage**				0.546			0.154
T1+T2	30	25	5		19	11	
T3+T4	66	58	8		51	15	
**pN stage**				0.787			0.961
pN0	41	35	6		30	11	
pN1	55	48	7		40	15	
**Grade of risk factors**				0.186			0.661
Moderate	6	4	2		4	2	
High	90	79	11		66	24	

## Discussion

In recent years, research into molecular targeted therapy of cancer has become a hot topic in the field of cancer research. The PD-1/PD-L1 pathway is involved in the occurrence and development of cancers, which induces effector T cell apoptosis, inhibits T cell activation and suppresses the body’s anti-tumour immune response [11]. As important members of the B7 family, PD-1/PD-L1 are expressed in a variety of tumour tissues. Overexpressed PD-L1 in tumour tissues was reported to downregulate anti-tumour effects by binding to its receptor PD-1. In the prostate cancer microenvironment, the overexpressed PD-L1 on APC cells can promote the growth of tumour cells and induce the death of related T lymphocytes with anticancer effects. In addition, the interaction between PD-1 and PD-L1 can inhibit the growth of T lymphocytes and the secretion of related anti-tumour factors [12]. Specific antibodies bind to PD-1 or PD-L1, blocking the PD-1 pathway to reactivate T cells; proliferate; and then enhance the anti-tumour immunity. Therefore, significant anticancer effects of the anti-PD-1 and anti-PD-L1 antibodies by blocking the resistance of PD-1/PD-L1 signalling pathways have been shown in many clinical trials [13, 14]. Several reports have described the increased expression of PD-1/PD-L1 in several tumours, such as breast, ovarian and oesophageal cancer [15–17]. In our study, the positive rates of PD-L1 expression in epithelial cells and lymphocytes between prostate cancer and benign prostatic tissues were 25.0% vs. 4.5% and 27.1% vs. 11.4%, respectively. Thus, the positive rate of PD-L1 in epithelial cells (*P* = 0.008) and lymphocytes (*P* = 0.038) was significantly higher in cancer than in benign tissues. No significant difference in PD-1-positive expression was found between cancer cases and benign tissues.

In line with previous findings, among the clinicopathological variables, the expression of PD-L1 in our results was related to Gleason score, but not to age, PSA level, lymph node metastasis, clinical stage or risk factor grade. In previous studies, a significant association of PD-L1 expression with adverse clinicopathological characteristics like higher PSA levels in prostate cancer was identified. For example, Gevensleben et al. revealed that clinicopathological features including proliferation, Gleason score and androgen receptor (AR) expression showed a positive association with moderate to high PD-L1 expression levels [18]. Meanwhile, in 130 untreated African American ethnicity prostate cancers, Calagua et al. revealed that PD-L1 positivity was prognostic for biochemical recurrence. Furthermore, the elevated serum PSA and small prostate independently predicted tumour PD-L1 positivity [19], whereas other reports showed different results and no significant association between PD-1/PD-L1expression and patient characteristics including the Gleason score, PSA, clinical TNM stage and pathological. TNM stage was shown [20].

Many items indicated that some genes or models including PD-1/PD-L1 had a certain correlation with prognosis in severe tumours [21–25]. For PD-1/PD-L1, Peng et al. conducted a systematic search to show the PD-L1 might be a predictive biomarker for EGFR-mutant non-small cell lung cancer treated with EGFR-TKIs [26]. Nomi et al. [27] found that PD-L1 expression was negatively correlated with lymphocytes in pancreatic cancer cells, especially tumour-infiltrating CD8+ T lymphocytes, these patients with positive PD-L1 expression often had a worse prognosis. Moreover, Ness et al. revealed a high density of CD8+ lymphocytes is an independent negative prognostic factor for biochemical failure-free survival [28]. Richardsen et al. revealed that a high expression of CD3+ lymphocytes in prostatic cancer tissue correlated with metastatic disease [29].

## Conclusion

In summary, our results revealed that the PD-L1-positive expression was significantly higher in cancer cases compared with benign tissues. No difference was found in PD-1-positive expression. In addition, PD-L1 was related to Gleason score and might be one co-factor that is associated with the progression of prostate cancer. However, our study was performed retrospectively in a single institution with a relatively small number of patients. Further studies with larger sample sizes and multicentre populations are necessary to confirm the results of this study. The associations of PD-L1 expression in prostate cancer with biochemical and clinical failure-free survival will be our next study.

## Data Availability

All data generated or analysed during this study are included in this published article.

## Abbreviations

PD-1: Programmed death 1
PD-L1: Programmed death ligand 1
mCRPC: Metastatic castration-resistant prostate cancer
pN: Pathologic nodal stage
pT: Pathologic tumour stage
PSA: Prostate-specific antigen
BPH: Benign prostatic hyperplasia
